# The effect of anxiety on emotional recognition: evidence from an ERP study

**DOI:** 10.1038/s41598-018-34289-8

**Published:** 2018-11-01

**Authors:** Qianqian Yu, Qian Zhuang, Bo Wang, Xingze Liu, Guang Zhao, Meng Zhang

**Affiliations:** 1 Research Center of Brain and Cognitive neuroscience, Liaoning Normal University, Dalian, 116029 China; 2 Department of Psychology, Xinxiang Medical University, Henan, 453003 China; 3 College of Public Health, Zhengzhou University, Henan, 450001 China

## Abstract

Anxiety-related bias in the recognition memory based on trait anxiety has induced some studies. Their results, however, were conflicting. In fact, anxious differences not only differed from personality traits but also from different anxiety mood levels. We explored the emotional memory bias in both trait and state anxiety individuals, the high trait and high state anxiety group, the high trait and low state anxiety group, the low trait and high state anxiety group, and the low trait and low state anxiety group, on classic recognition paradigm using event-related potentials (ERPs). The behavioral results showed high state anxiety levels increased the *d’* of negative words, regardless of the trait anxiety of participant is high or low, and a lower *d’* of recognition memory for negative words than for neutral and positive words in all participants. Moreover, Electrophysiological results supported the findings of behavior, showing an earlier N400 (250–500 ms) latency elicited for new-negative words in high state level than in low state levels in right parietal region. These results suggested that the memory bias to negative events resides in state anxiety, but not in trait anxiety.

## Introduction

Anxiety is anticipation of future threat, it’s more often associated with muscle tension and vigilance in preparation for future danger and cautious or avoidant behaviors, some the level of anxiety is reduced by pervasive avoidance behaviors^1,2^. There has been a large increase in the incidence rates of anxiety disorders and symptoms over the years, however, it is hard to investigate anxiety independently due to the high comorbidity rate (as much as 50%)^3^. Trait anxiety, innate predisposition, is a relatively stable personality trait with the reaction of the individual differences^4^. Studies found that high trait anxiety of human was extremely similar with anxiety disorders about behavioral and cognitive features, so individuals with high trait anxiety from a non-clinical group have a predisposition for an anxiety-related bias in emotional and cognitive processing, high trait anxiety is considered to be a prerequisite for psychiatric disorders^5,6^. In other words, individuals with high trait anxiety may be a proper sample to uncover the characters of anxiety, understanding the neural basis in non-clinically anxious individuals will provide a broad picture of the neuro-scientific basis of anxiety disorders^7^.

Cognitive bias, especially the memory processing, were the focus of many researchers, whereas the evidence for anxiety-related recognition memory bias is still scant and inconsistent across studies^8–12^, some studies showed a bias to recognize threatening items^13–15^, some got a result contrary^16–20^. Up to now, whether there has a robust bias in trait anxiety under the negative recognition processing is still unclear. Cognitive-motivational view consider the effect of negative biases in anxiety is not only trait anxiety, but also have situational context, state anxiety, prior learning, biological preparedness^21^. And model of William *et al*. representing the cognitive mechanisms underlying biases in initial orienting to treat in anxiety is interaction effect of state and trait anxiety on attentional bias^22^. Anxiety can be classified as trait anxiety and state anxiety^23,24^. State anxiety is a common mood in daily life, and it can be a kind of brief emotional experience, accompanied by physiological arousal and subjective feelings (e.g., nervous, fear) as environmental change^4^. Specifically, the correlation is very high between trait and state anxiety, many studies had found that the effect of state anxiety was stronger than trait anxiety to task performance^25–27^, furthermore, a behavioral research found that the effect of recognition negative biases was state anxiety alone, not trait anxiety^12^. The factor, state anxiety levels (e.g. high vs. low), might affect the size and direction of threat bias in recognition tasks^21,28^. Thus, the present study intends to control the state anxiety level into high and low state anxiety groups by experiment operation. Since in most studies state anxiety has more significant impact on task performance than trait anxiety^25–27,29^, we predicted state anxiety would have the most obvious infection on memory bias than trait anxiety.

Therefore, in order to explore whether individuals with high trait anxiety and state anxiety have anxiety recognition bias, we employed a classic recognition paradigm, used the neutral, positive and negative words as stimulus. We added experimental operation between learning and testing phase to induce/calm state anxiety, the experimental operation was that participants were asked to read one of two scenarios describing different events and then image that they were experiencing the event^29–31^, what’s more, studies have showed that this experimental operation was very effective in inducing state anxiety (negative mood states)^27,30–32^. Thence, participants were divided into high state anxiety group and low state anxiety group by the experiment operation. Meanwhile, participants were also divided into high and low trait group according to their trait anxiety scores. Altogether, four groups (high state with high trait group, high state with low trait group, low state with high trait group and low state with low trait group) were involve in the present study. Given the stronger effect of state anxiety than trait anxiety to task performance^25–27^, and relatively stable personality of the trait anxiety, high trait individuals might induce higher state anxiety compared to low trait anxiety individuals, we predicted state anxiety is an intervening variable between trait anxiety and emotional valence events. If there existed anxiety recognition bias, the high state with high trait group would obtain the maximized effect, followed by the high state with low anxiety group, low state with high anxiety group, low state with low trait anxiety. Further, event-related potential (ERP) possess excellent temporal resolution and a continuous measure of processing, thus, we collected participants’ electroencephalography (EEG) data synchronously. Many studies claimed that the N400 was related with semantic processing^33–35^, moreover, was influenced by the effects of emotional valence^36–38^, and, participants’ emotional state^36,39,40^. Thence, the index of parietal N400 might be sensitive and effective to emotional words in present study.

## Method

### Participants

Two hundred participants were selected by the random sampling method from five grades’ undergraduates, who took elective courses in psychology in Xinxiang Medical University. All the participants were tested by the Trait Anxiety Inventory (the second 20 questions of state - trait anxiety questionnaire)^41^. The T-AI consists of 20 self-report items that measure anxiety-related trait personality, with high internal consistency and test-reliability ranging from 0.73 to 0.86 across multiple samples^23,42^. The top and down 27% of the all trait anxiety score were selected for the study and the Chinese norm score of trait anxiety are 41.11 ± 7.74 (males) and 41.31 ± 7.54 (females) respectively^43^. Participants were recruited and divided into two groups with the following standard of evaluation: participants scored higher than or equal to 45 were assigned to the high trait anxiety group, while the ones scored lower than or equal to 40 were assigned to the low trait anxiety group. Seventy-four participants **(**38females; mean age = 20.45 ± 1.59 years**)** were recruited depending on the scores. They got the formal experiment in the laboratory of psychology. Altogether, thirty-seven participants (18males, 19females; mean score = 50.32 ± 5.34) were assigned to the high trait anxiety group, and thirty-seven participants (18males, 19females; mean score = 34.51 ± 4.63) were assigned to the low trait anxiety group and then an independent samples *t* test showed this difference to be significant *t* (72) = 0.35, *p* < 0.01. Three participants were excluded from further analysis because of noisy electroencephalography. Thence, the induced state anxiety group consisted of 20 high trait anxiety participants (10 males, 10 females) and 17 low trait anxiety participants (7 males, 10 females); the non-induced state anxiety group consisted of 16 high trait anxiety participants (7 males, 9 females) and 16 low trait anxiety participants (9 males, 7 females). All participants were right-handed, and have normal or corrected-to-normal vision.

### Ethics Statement

All participants gave their written informed consent in accordance with the Declaration of Helsinki^44^. The ethics committee of the Xinxiang Medical University approved this study. The experimental methods were carried out in “accordance” with the approved guidelines.

### Materials

#### Materials

Stimulus were selected from Chinese Affective Words System(CAWS)(Wang & Zhou, 2008). There were 60 negative-arousing words (mean valence = 2.54; mean arousal = 6.63), 60 positive-arousing words (mean valence = 7.35; mean arousal = 6.61), and 60 neutral, non-arousing words (mean valence = 5.40; mean arousal = 4.13). Altogether, one hundred eighty words were included in the experiment. Word frequencies were equated across emotion.

#### Procedure

The experiment was composed of three phases, one study phase, one imagining phase and one test phase. In the study phase, participants were instructed to memory 120 words (40 neutral, 40 negative, 40 positive). Each of the words was displayed for 1500 ms. Then in imagining phase, the instruction 1 was presented to the induced state anxiety group, or the instruction 2 was presented to the non-induced state anxiety group. Before and after the imagining phase, participants were tested by the State-Trait Anxiety Inventory (the first 20 questions of state-trait anxiety questionnaire), it was used to measure participants’ state anxiety level. Immediately following the presentation of the study list, participants made recognition judgments for 120 words, which 60 studied words that had been presented in the study phase, and 60 new words had never been presented before, the words were presented one at a time for 1000 ms each, then the response interface was presented for 2000 ms to made participants delay to respond. All stimuli were presented in white letters against black backgrounds. For items judged to be “old” pressing “F” key, lures judged to be “new” by pressing “J” key. Study and test sequences were randomly ordered for each subject. The instruction 1 and instruction 2 were depicted as follows:

Instruction 1: Hello, welcome to participate in this study. This is a test to inspect the ability of imagination about details.

Now please image that: you got a call from you tutor suddenly, the tutor asked you to go to his office at once, it seems that there will be a very serious matter. Please image all the details and your psychological physiology reaction between the period you got the call and you got to the office.

Please press the space bar to start the test when you understand the introduction.

Instruction 2: Hello, welcome to participate in this study. This is a test to inspect the ability of imagination about details. Now please recall all the details that you got out of the bed and washed in this morning.

Please press the space bar to start the test when you understand the introduction.

#### ERP data acquisition

Brain electrical activity was recorded from 64 Ag-AgCl scalp sites according to the international 10–20 system in an elastic cap (NeuroScan Prouct). During recording, all electrodes were referenced to Cz and re-referenced off-line to linked mastoids. Channels for horizontal and vertical EOG were computed offline from electrodes recorded from the outer canthi of the eyes and from above and below the right eye, respectively. Electrode impedance was kept below 5 kΩ.

EEG was sampled on-line with a frequency of 500 Hz DC-amplifiers with a band-pass filter of 0.1–100 Hz. Data was filtered off-line by a band-pass filter of 0.1–25 Hz and runned an independent component analysis(ICA) for eye movement correction^45^.

### Data analysis

#### Behavioral data analysis

A standard way of measuring accuracy is to calculate β and *d’*, the distance between the means of the memory strength distributions of studied words and unstudied words. The percentages of correct answers on the memory test were subjected to three-way repetitive measure analysis of variance (ANOVA). We applied the theory of detection to the memory test.

According to the signal detection theory (SDT), we defined four possible outcomes for each trial depending on a word of object chosen from the study phase or not (old/new judgement) and participants’ response: hits, misses, false alarms (FA), and correct rejections (CR). Specifically, a hit is an accurate judgement that word has been shown in the study phase (old word); a miss is a failure detection for the old word; a correct rejection is an accurate judgement that the word was not chosen in the study phase; and a false alarm is a failure detection for the new word. Then the P (H) and the P (FA) in the items and the lures were analyzed by the following formula:

$$P({\rm{H}})=n({\rm{H}}{\rm{I}}{\rm{T}})/({\rm{n}}({\rm{H}}{\rm{I}}{\rm{T}})+{\rm{n}}({\rm{M}}{\rm{I}}{\rm{S}}{\rm{S}}))$$

$${\rm{P}}({\rm{FA}})={\rm{n}}({\rm{FA}})/({\rm{n}}({\rm{FA}})+{\rm{n}}({\rm{CR}}))$$

The P(H) and the P(FA) in the items and the lures were translated to O(H), O(FA) Z(H) and Z(FA) using PZO translation. Then, the likelihood ratio (β) and discriminability index (*d’*) in the items and the lures were analyzed by the following formula:

$${\rm{\beta }}={\rm{O}}({\rm{H}})/{\rm{O}}({\rm{FA}})$$

$$d\mbox{'}={\rm{Z}}({\rm{H}})-{\rm{Z}}({\rm{FA}})$$

Higher β values (the likelihood ratio or decision criteria, the more the β is, the more strict the criteria is) indicated worse memory performance in this study. Higher *d’* values (discriminability index or sensibility, the more the *d’* is, the more sensibility is) indicates best memory performance in this study. The β and *d’* values in the two conditions were subjected to three-way ANOVA. All *p*-values were corrected using the Bonferroni adjustment.

#### ERP data analysis

ERPs were calculated time-locked to the onset of the search display, with segments extending from 100 ms before stimulus onset until 1000 ms afterwards. In analyses, the 100 ms interval preceding target onset served as baseline. Artifacts produced by blinks or eye movements were corrected by subtracting means of ICAs implemented in the EEGLab software^45^. Trials with incorrect responses were excluded from analysis.

### Significance

Studies found that anxiety-related recognition memory bias was still inconsistent. Here, we asked different trait anxiety level individuals under different state anxiety levels to perform classic learn-test task. Our results showed that there existed the memory bias to negative events in state anxiety, but not in trait anxiety.

## Results

### Manipulation check

The pretest and posttest’s scores of STAI-S of the induced state anxiety groups were calculated separately (pretest mean = 38.85 ± 9.16; posttest mean = 41.28 ± 11.85), and then a paired sample t test showed this difference was significant *t* (38) = −2.10, *p* < 0.05. Similarly, the twice scores of STAI-S of the calm state anxiety groups were calculated separately (pretest mean = 36.19 ± 6.99; posttest mean = 36.63 ± 7.61), and the result of paired sample t test showed there was no difference *t* (31) = −0.54, *p* > 0.1. Therefore, the experiment manipulation is effective.

### Behavioral data

1. *d’*. A 2 (state anxiety: high vs. low) × 2 (trait anxiety: high vs. low) × 3 (emotional valence: negative vs positive vs neutral) repeated-measure ANOVA was performed. The interaction between valence and state anxiety was significant, F (2, 66) = 6.048, *p* < 0.01, η^2^ = 0.083. Subsequent simple effect analyses showed that significant differences between high state anxiety group and calm group about the *d’* were found of negative words (F (1, 67) = 20.16, *p* < 0.01), but not in neutral and positive words (*p*s > 0.1), showing that the memory recognition processing was different to negative words and the other two groups’ words. In addition, state anxiety influenced memory retrieval phase on recognition task, but trait anxiety did not. Moreover, only the *d’* of negative words of high state anxiety group was larger than calm group. The results showed that there was significant main effect of emotional valence, F (2, 66) = 55.66, *p* < 0.001, η^2^ = 0.454, and state anxiety, F (1, 67) = 7.34, *p* < 0.01, η^2^ = 0.10. There was no significant difference between positive words and neutral words, but the *d*’ of negative words was smaller than positive words and neutral words. No other statistical differences were found (all *p*s > 0.1). Table 1 Mean accuracy (%) of trait, state anxiety and emotional valence for all conditions above. Table 2. Mean *d’* of trait, state anxiety and emotional valence for all conditions above.

**Table 1:** Mean accuracy (%) of trait, state anxiety and emotional valence for all conditions above.

	Negative	Neutral	Positive
induced state and high trait anxiety	0.62 ± 0.09	0.66 ± 0.08	0.68 ± 0.10
induced state and low trait anxiety	0.61 ± 0.09	0.70 ± 0.07	0.69 ± 0.07
non-induced state and high trait anxiety	0.62 ± 0.07	0.67 ± 0.06	0.70 ± 0.07
non-induced state and low trait anxiety	0.61 ± 0.11	0.66 ± 0.07	0.66 ± 0.08

**Table 2:** Mean β and *d’* of trait, state anxiety and emotional valence for all conditions above.

	Negative		Neutral		Positive	
	β	d’	β	d’	β	d’
induced state and high trait anxiety	1.11 ± 0.30	0.63 ± 0.52	1.12 ± 0.68	1.21 ± 0.72	1.15 ± 0.72	1.00 ± 0.51
induced state and low trait anxiety	1.04 ± 0.21	0.53 ± 0.59	1.36 ± 0.76	1.09 ± 0.71	1.26 ± 0.44	1.23 ± 0.45
non-induced state and high trait anxiety	1.26 ± 0.76	−0.03 ± 0.62	1.36 ± 0.71	1.21 ± 0.50	1.25 ± 0.57	1.03 ± 0.27
non-induced state and low trait anxiety	1.14 ± 0.44	−0.01 ± 0.45	1.29 ± 0.75	0.96 ± 0.63	1.08 ± 0.49	1.09 ± 0.53
2. β. B 2 (state anxiety: high vs. low) × 2 (trait anxiety: high vs. low) × 3 (emotional valence: negative vs positive vs neutral) repeated-measure ANOVA was performed. We have not found any differences about β. Table 2. Mean β of trait, state anxiety and emotional valence for all conditions above.

### ERP data

#### N400 (250–500 ms timing window)

According to previous researches, N400 was related to memory^46,47^ and semantic processing^33–35^, and even to the emotional valence^36–38^ and participants’ emotional state^36,39,40^. Here, we used the parietal N400 as an index on recognition memory. The latency and peak of the N400 were measured from 250 to 500 ms, after the stimulus onset, as defined in previous researches^48–51^, centroparietal (CPz, CP1/2, CP3/4, CP5/6) and parietal (Pz, P1/2, P3/4, P5/6), latencies and peaks of typically maximum amplitude regions for N400 were analyzed.

For the latencies of N400, a 2 (trait anxiety: high/low) × 2 (state anxiety: high/low) × 3 (emotional valence: negative/neutral/positive) × 2 (responses: old/new) repeated measures ANOVA were analyze. The interactions of valence, response and state anxiety were significant over right parietal (i.e., P4) and centroparietal (i.e., CP1/CPZ/CP2/CP4/CP6). There are significances about the interaction of response, trait and state anxiety over right parietal (i.e., P1/P4) and centroparietal (i.e., CP1/CPZ/CP2/CP4/CP6). The other main effects and interactions were not significant or just significant in few sites, so no further analysis were carried out on them. The specific data were affixed in Table 3.

**Table 3:** The latencies of N400 were analyzed with a 2 (trait anxiety: high/low) × 2 (state anxiety: high/low) × 3 (emotional valence: negative/neutral/positive) × 2 (responses: old/new) repeated measures ANOVA.

	df	CP1		CPZ		CP2		CP4		CP6		P1		P4	
		F	η^2^	F	η^2^	F	η^2^	F	η^2^	F	η^2^	F	η^2^	F	η^2^
responses	1, 67	1.72	0.03	3.25	0.05	1.02	0.02	0.03	0.01	0.70	0.01	0.19	0.01	0.14	0.01
responses*trait	1, 67	1.41	0.02	0.02	0.01	2.14	0.03	2.65	0.04	2.06	0.03	0.01	0.01	0.70	0.01
responses*state	1, 67	2.09	0.03	0.76	0.01	2.14	0.03	0.26	0.01	0.03	0.01	0.06	0.01	1.17	0.02
responses*trait*state	1, 67	7.78**	0.10	6.61*	0.09	6.10*	0.08	10.70**	0.14	4.07*	0.06	9.18**	0.12	6.53*	0.09
emotion	2, 66	1.70	0.25	2.22	0.03	4.14*	0.06	1.46	0.02	0.93	0.01	6.59**	0.09	0.93	0.01
emotion*trait	2, 66	0.21	0.01	0.60	0.01	0.79	0.01	0.52	0.01	0.21	0.01	0.82	0.01	0.24	0.21
emotion*state	2, 66	0.50	0.01	2.14	0.03	0.66	0.01	0.25	0.01	1.53	0.02	0.17	0.01	0.60	0.01
emotion*trait*state	2, 66	1.19	0.02	1.44	0.02	1.13	0.02	0.87	0.01	2.46	0.04	0.78	0.01	0.37	0.01
responses*emotion	2, 66	0.20	0.03	0.69	0.01	0.45	0.01	2.06	0.03	1.90	0.03	0.58	0.01	1.68	0.02
responses*emotion*trait	2, 66	2.55	0.04	1.15	0.02	0.20	0.01	0.46	0.01	0.31	0.01	1.10	0.02	1.03	0.02
responses*emotion*state	2, 66	4.80*	0.07	6.27**	0.09	7.18**	0.10	4.55*	0.06	4.50*	0.06	2.33	0.34	4.85*	0.07
response*emotion*trait*state	2, 66	0.61	0.01	0.23	0.01	0.14	0.01	1.00	0.02	0.39	0.01	0.56	0.01	0.15	0.02
trait	1, 67	0.01	0.01	0.03	0.01	0.00	0.00	0.32	0.01	0.01	0.01	0.08	0.01	0.22	0.01
state	1, 67	0.25	0.01	0.23	0.01	0.10	0.01	0.10	0.01	1.06	0.02	1.41	0.02	1.45	0.02
trait*state	1, 67	1.16	0.02	0.13	0.01	0.34	0.01	1.45	0.02	0.71	0.01	1.85	0.03	2.09	0.03

*Notes:* *<0.05, **<0.01.

**Figure 1:**
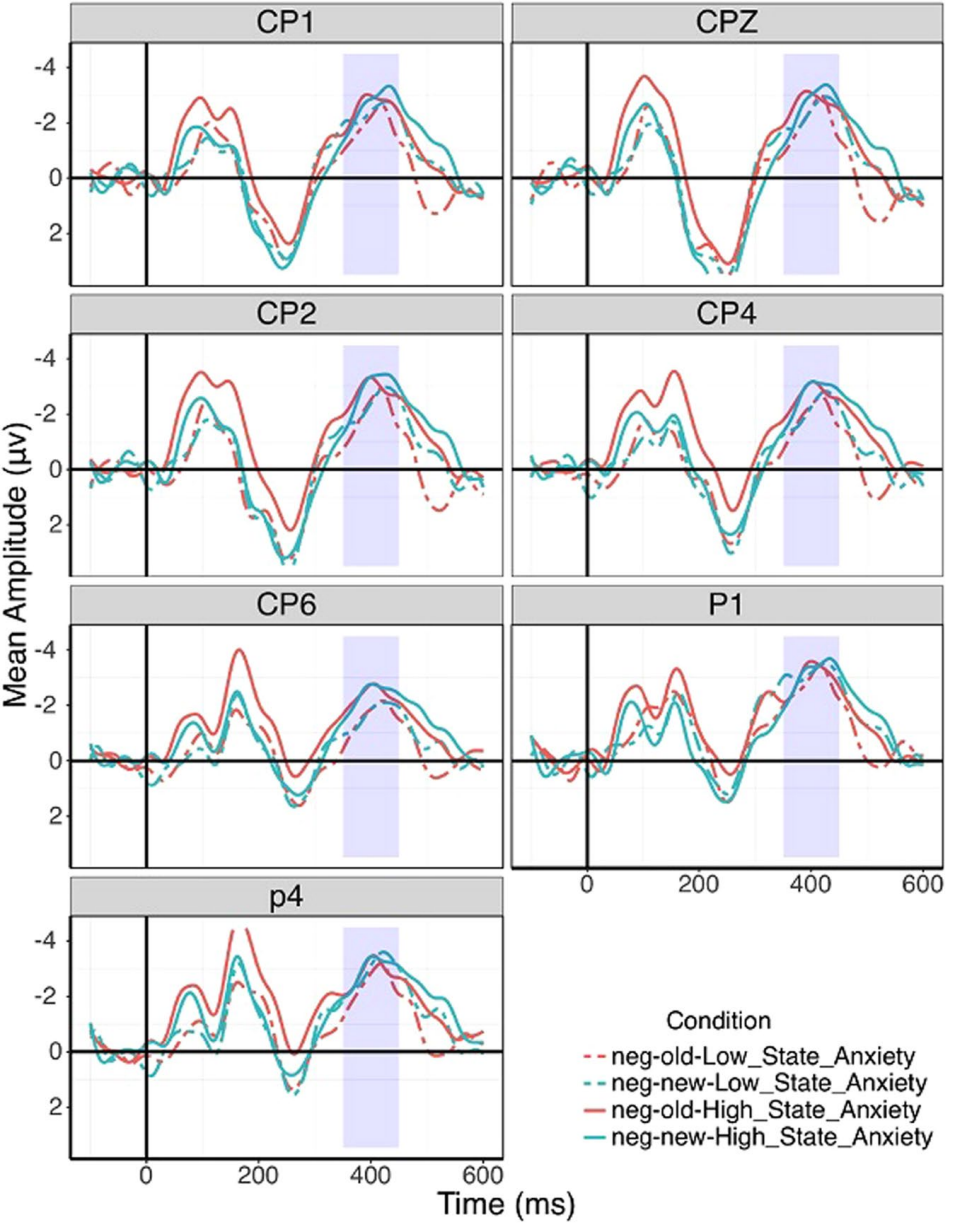
The separate N400s of above 7 electrode sites for all four conditions to negative words.

For the interaction of valence, response and state anxiety, we reanalyzed it with a 2 (state anxiety: high/low) × 3 (emotional valence: negative/neutral/positive) × 2 (responses: old/new) repeated measures ANOVA. There were significances about the interaction of valence, response and state anxiety over right parietal (CP1: F(2, 68) = 4.72, *p* < 0.05, η^2^ = 0.06; CPZ: F(2, 68) = 6.37, *p* < 0.01, η^2^ = 0.08; CP2: F(2, 68) = 7.40, *p* < 0.01, η^2^ = 0.10; CP4: F(2, 68) = 4.60, *p* < 0.05, η^2^ = 0.06; CP6: F(2, 68) = 4.64, *p* < 0.05, η^2^ = 0.06; P4: F(2, 68) = 4.94, *p* < 0.05, η^2^ = 0.07). No other statistical differences were found (all *p*s > 0.1). Subsequent simple main effect tests showed that the latency induced by negative words was shorter than calm group under the new words condition in anxious state group (CP1: F(1, 69) = 5.86, *p* < 0.05, η^2^ = 0.08; CPZ: F(1, 69) = 10.20, *p* < 0.01, η^2^ = 0.13; CP2: F(1, 69) = 6.77, *p* < 0.05, η^2^ = 0.09; CP4: F(1, 69) = 3.55, *p* < 0.1, η^2^ = 0.05; CP6: F(1, 69) = 7.29, *p* < 0.01, η^2^ = 0.10; P4: F(1, 69) = 5.46, *p* < 0.05, η^2^ = 0.07), there was no same differences among other parts. For the interaction of response, trait and state anxiety, we reanalyzed it with a 2 (state anxiety: high/low) × 3 (trait anxiety: high/low) × 2 (responses: old/new) repeated measures ANOVA. There were significances about the interaction of response, trait and state anxiety over parietal (CP1: F(1, 67) = 7.78, *p* < 0.01, η^2^ = 0.10; CPZ: F(1, 67) = 6.61, *p* < 0.05, η^2^ = 0.09; CP2: F(1, 67) = 6.10, *p* < 0.05, η^2^ = 0.08; CP4: F(1, 67) = 10.69, *p* < 0.01, η^2^ = 0.14; CP6: F(1, 67) = 4.07, *p* < 0.05, η^2^ = 0.06; P1: F(1, 67) = 9.18, *p* < 0.05, η^2^ = 0.12; P4: F(1, 67) = 5.11, *p* < 0.05, η^2^ = 0.07). No other statistical differences were found (all *p*s > 0.1). Subsequent simple main effect tests showed that the latency induced by new words was shorter than calm group in high trait anxiety group(CP1: F(1, 67) = 5.17, *p* < 0.05, η^2^ = 0.07; CP2: F(1, 67) = 3.99, *p* < 0.1, η^2^ = 0.06; CP4: F(1, 67) = 4.84, *p* < 0.05, η^2^ = 0.07; CP6: F(1, 67) = 4.00, *p* < 0.1, η^2^ = 0.07; P1: F(1, 67) = 5.96, *p* < 0.05, η^2^ = 0.08; P4: F(1, 67) = 6.05, *p* < 0.05, η^2^ = 0.08), there was no same differences among other parts. Figure 1. Mean N400s of above 7 electrode sites for all four conditions to negative words.

The peaks of N400 were analyzed with a 2 (trait anxiety: high/low) × 2 (state anxiety: high/low) × 3 (emotional valence: negative/neutral/positive) × 2 (responses: old/new) repeated measures ANOVA. The results showed that there was significant main effect of response, the amplitude of new word was stronger than old word (CP5: F(1, 67) = 4.06, *p* < 0.05, η^2^ = 0.06; CP3: F(1, 67) = 3.84, *p* < 0.1, η^2^ = 0.04; CP1: F(1, 67) = 5.39, *p* < 0.05, η^2^ = 0.07; CPz: F(1, 67) = 5.03, *p* < 0.05, η^2^ = 0.07; CP2: F(1, 67) = 6.25, *p* < 0.05, η^2^ = 0.09; CP4: F(1, 67) = 6.75, *p* < 0.05, η^2^ = 0.09; CP6: F(1, 67) = 3.39, *p* < 0.1, η^2^ = 0.05; P5: F(1, 67) = 6.49, *p* < 0.05, η^2^ = 0.08; P3: F(1, 67) = 3.66, *p* < 0.1, η^2^ = 0.05; P1: F(1, 67) = 4.20, *p* < 0.05, η^2^ = 0.06; Pz: F(1, 67) = 2.84, *p* < 0.1, η^2^ = 0.04; P2: F(1, 67) = 4.82, *p* < 0.05, η^2^ = 0.07; P4: F(1, 67) = 4.19, *p* < 0.05, η^2^ = 0.06; P6: F (1, 67) = 3.57, *p* < 0.1, η^2^ = 0.05), there was no same differences among other parts. The result, ERPs of words correctly judged as ‘new’ have been observed to be more negative-going than ERPs elicited by words correctly judged as “old”, named “old/new effect”, accorded with previous research results in recognition memory task^52–55^.

## Discussion

The aim of the present study was to investigate whether anxious individuals have memory bias to such negative events. Participants’ electroencephalography brain activities and responses were recorded while they were performing a classic recognition task. The results showed high state anxiety levels increased the *d’* of negative words, moreover, an earlier N400 (250–500 ms) latency was elicited for new-negative words in high state levels than in low state levels in right parietal region, which suggested there existed the recognition memory bias to negative events in state anxiety (mood factor). Trait anxiety (personality factor), however, did not influence recognition memory.

Notably, the *d’* of negative words of high state anxiety individuals was larger than that of low state anxiety individuals, whether it’s high or low trait anxiety individuals in the present study. A higher d’ indicates that the signal can be more readily detected, *d’* depends on the intensity (i.e., the physical properties of the stimulus materials) and the sensitivity of the individual, and the sensitivity of the individual is affected by his/her pre se ability and physiological state^56,57^. Considering that the increasing state anxiety level might induce the vulnerable of negative information processing, which reflected in the change of d’, high state anxiety individuals may have a stronger vigilance of threatening information than low state anxiety individuals. Furthermore, the result also showed there did not exist significant difference in β, which suggested that subjects’ subjective motivation was similar for the three types of words, and this result support to no negative response bias existed. Moreover, the *d’* of recognition memory was lower for negative words than both neutral and positive words in all participants. The data indicated a significant emotional valence effect, many studies reported that the false recognition of negative stimuli was higher than of the neutral stimuli^58–60^, which consistent to our results that the participants’ ability of distinguishing new and old negative words was poorer than that of neutral and positive words, healthy individuals would enhance negative affective processing, meanwhile, they also have the capacity away from negative processing, finally, there still existed greater d’ on positive and neutral words relative to negative words. State anxiety increasing may induce the negative memory bias in the present study.

Moreover, we found that the evidence in ERP data supported the finding of behavior. It is interesting to note that the enhancement was found in the new negative words, that the latency of N400 of new negative words in high state levels was shorter than that in low state levels in right parietal region. It suggested that state anxiety influenced the new negative words but not old negative words. Many studies have claimed that the latency of N400 was related with semantic processing^61–64^, moreover, many studies had found that N400 was influenced by the effects of emotional valence^36–38^, and the effect of participants’ emotional state^36,39,40^. So, the index of parietal N400 was sensitive and effective to emotional words in different state anxiety levels in present study. Here, the latency of N400 was the index that indicated memory recollection of semantics of words, and the result suggested the latency of N400 was influenced by the effect of emotional valence of words and state anxiety levels. As for we found the difference of latency only in right parietal region, we considered that it is due to the lateralization of emotion process, convincing evidence had indicated that the left hemisphere is dominant for positive emotions and the right hemisphere is dominant for negative emotions^65–69^. Hence, our results that the difference of ERP data of the negative words in right parietal region were consistent with previous studies.

Meanwhile, the result also showed that in high trait anxiety participants, the latency of new words in induced state anxiety group was shorter than calm group, but there was no significance in emotional valence. Thus, it might become another evidence that there did not existed negative recognition biases in trait anxiety individuals. The difference had not been found in low trait anxiety group, there might exist stronger state anxiety level in high trait anxiety group than low trait anxiety group, there might really exist individual difference between high and low trait anxiety individuals, but none in recognition memory. It found more vigilance in high state anxiety than low state anxiety level in high trait anxiety individuals. In addition, in the present study, the peak of N400s were found the “old/new” effect, ERPs of words correctly reject have been observed to be more negative-going than ERPs elicited by words hit, was consistent with previous research results in recognition memory task^52–55^, nevertheless, there was no difference in trait and state anxiety levels, which may imply there was no difference for the degree of semantic processing both trait and state anxiety, the finding need to be further examined

On the whole, the behavioral outcome reflected state anxiety affected only negative words on memory retrieval phase, further, the physiological results suggested that state anxiety affected only the new words instead of old words about negative words, the effect was accorded with previous studies, which found the difference was in new words and not old words^52,70,71^. According to vigilance-avoidance hypothesis^72–77^, vigilance for negative events could enhance succeeding memory trace of the negative event for anxious individuals. Anxious individuals always more preferentially attend to negative events, encode them more deeply and retrieve memories more easily and correctly, as it could make anxious individuals considering the current situation more negative than it actually was. However, it has been shown that anxious individuals could avoid a prolonged or deep processing of negative information^78^, which lessened the number of rehearsals during the coding stage or limited retrieval of negative information. Therefore, the effect of vigilance could be reduced or even overturned by the effect of avoidance. In this respect, for the memory of a negative events of anxious individuals, hyper-vigilance and avoidance may conflict with each other. In the present study, the results showed the effect of avoidance might be stronger than the effect of hyper-vigilance, but the vigilance to negative information of high state anxiety individuals was stronger than of which low state anxiety individuals. State anxiety still leaded to recognition memory bias, but trait anxiety did not. Further, from an adaptive perspective, such anxiety enhances negative processing, thus increases vigilance level against danger in a threatening environment^79,80^, notably, increases vigilance towards novels stimuli^81^. In low state anxiety levels, healthy individuals may tend to avoid, sometimes can act in “blissful ignorance”^82^. For example, it may be that inhibiting the processing of new negative words processing (and, hence, making more errors in response to negative information) is adaptive. In fact, to process all stressors in a constantly changing but remains safety environment would be cognitively unnecessary and wasteful. As such, “blissful ignorance,” or an avoidance to fearful stimuli, may increase adaptive fitness under low state anxiety levels conditions^83^. However, in high state anxiety levels, this situation reversed, such that it becomes adaptive to focus on all aversive information in order is to avoid harm, it also means that enhance vigilance for negative stimulus to avoid harm. As predicting, the effect of avoidance was still stronger than the effect of hyper-vigilance, there was no real threat to participants in experiment in inducing state anxiety condition. But, relative to the condition of low state anxiety levels, subjects in high state anxiety levels could serve the purpose of increasing vigilance to new negative words, could better distinguish to the new or old judge of negative words. State anxiety would still lead to recognition memory bias.

## Conclusion

Individuals with high trait anxiety form a non-clinical group with a predisposition for an emotional and cognitive processing bias that is considered to be a pre-existing condition for psychiatric disorders. Our results showed high trait anxiety individuals had no recognition memory bias, but, individuals with high state anxiety existed recognition memory bias to negative events. The finding recognition memory was influenced by state anxiety, rather than trait anxiety. There still existed limitation that whether encoding stage was influenced by state and trait anxiety levels. There is an adaptive perspective that, in low state anxiety levels, individuals inhibit negative processing. But in high state anxiety levels, individuals could increase vigilance to new-negative words to avoid potential threat, enhancing distinguish the new/old judgement of negative words.

## Data Availability

The datasets generated during and/or analyzed during the current study are available from the corresponding author on reasonable request.
